# Increased Mortality in Diabetic Foot Ulcer Patients: The Significance of Ulcer Type

**DOI:** 10.1155/2016/2879809

**Published:** 2016-04-24

**Authors:** N. K. Chammas, R. L. R. Hill, M. E. Edmonds

**Affiliations:** ^1^Diabetic Foot Clinic, King's College Hospital, Denmark Hill, London SE5 9RS, UK; ^2^Department of Medical Microbiology, King's College School of Medicine, King's Denmark Hill Campus, Bessemer Road, London SE5 9PJ, UK

## Abstract

Diabetic foot ulcer (DFU) patients have a greater than twofold increase in mortality compared with nonulcerated diabetic patients. We investigated (a) cause of death in DFU patients, (b) age at death, and (c) relationship between cause of death and ulcer type. This was an eleven-year retrospective study on DFU patients who attended King's College Hospital Foot Clinic and subsequently died. A control group of nonulcerated diabetic patients was matched for age and type of diabetes mellitus. The cause of death was identified from death certificates (DC) and postmortem (PM) examinations. There were 243 DFU patient deaths during this period. Ischaemic heart disease (IHD) was the major cause of death in 62.5% on PM compared to 45.7% on DC. Mean age at death from IHD on PM was 5 years lower in DFU patients compared to controls (68.2 ± 8.7 years versus 73.1 ± 8.0 years, *P* = 0.015). IHD as a cause of death at PM was significantly linked to neuropathic foot ulcers (OR 3.064, 95% CI 1.003–9.366, and *P* = 0.049).* Conclusions.* IHD is the major cause of premature mortality in DFU patients with the neuropathic foot ulcer patients being at a greater risk.

## 1. Introduction

There is strong epidemiological evidence of excess mortality in association with the diabetic foot syndrome. There is a greater than twofold increase of mortality in diabetic foot ulcer (DFU) patients compared to nonulcerated diabetic patients, regardless of age, type and duration of diabetes, treatment of diabetes, glycated haemoglobin concentration, history of lower extremity amputation, and cumulative pack years of cigarette use [1]. Diabetic patients with leg and foot ulcers have a lower 5-year survival (43%) than nondiabetic ulcerated subjects (56%) and general population controls (68%) [2].

Reported mortality rates for diabetic foot ulcer (DFU) patients range from ≈10% on a median follow-up of 16 months [3] to 24% after 5 years [4]. A study from a Liverpool foot clinic indicated a 5-year mortality rate as high as 44% in patients presenting with new DFUs [5]. A large community based Norwegian study over a 10-year follow-up period reported an increased mortality of 49% in diabetic patients with a history of DFU compared with 35.2% of diabetic patients without a history of foot ulcers and 10.5% of nondiabetic individuals [6]. We previously reported preliminary results on our patients but only had information on 112 of 243 death certificates and 41 out of 80 postmortem examinations that were carried out [7]. The present paper has information on the full number of 243 death certificates and 80 postmortem examinations. The aim of this study was to establish the precise cause(s) of death in DFU patients and to examine the relationship between cause of death and ulcer type.

## 2. Materials and Methods

To delineate causes of death in DFU patients, we conducted an 11-year retrospective (death search) audit of all deceased subjects who attended the Diabetic Foot Clinic at King's College Hospital and whose records were available in this institution. Occurrence of death was confirmed from hospital medical notes and general practitioners' records. Information provided for each patient included full name, address, date of birth, date of death, age at death, and place of death.

Causes of death were established for deaths occurring between April 1989 and January 2000 from the following:

- death certificates obtained from the central register for England and Wales held in Southport through the Family Record Centre in London. In cases where death certificates could not be retrieved from the central register, individual registry offices of various boroughs of London were approached for those certificates;
- postmortem examination results as stated in hospital records and coroners' reports on death certificates.

Study subjects included type 1 and type 2 diabetes patients as per WHO classification (1997) (Table 1). DFU patients were stratified into two categories according to type of ulcer. Ischaemia was diagnosed by absence of foot pulses [8]. Neuropathy was determined by the presence of a stocking distribution of sensory loss to light touch (cotton wool). Patients with signs of neuropathy and palpable pulses were said to have neuropathic ulcers and patients with absent foot pulses with or without neuropathy were deemed to have ischaemic ulcers.

We sought a control group of diabetic patients who attended the diabetic clinic at King's College Hospital and died over a 10-year period from March 1990 to March 2000. These patients had diabetes mellitus but no history of foot ulceration. They were stratified to match the DFU group in age and type of diabetes. In the UK, the major immediate cause of death is classified as 1a, 1b, or 1c on the death certificate. Death from ischaemic heart disease (IHD) was defined as death due to coronary artery disease or fatal myocardial infarction from occlusive coronary thrombosis or secondary fatal dysrhythmias.

### 2.1. Statistical Analysis

Mean age at death was calculated, including its standard deviation, and significance was tested at 95% confidence interval. A *P* value of <0.05 was considered statistically significant. The significance of the risk of death from IHD in diabetic patients with neuropathic ulcers compared to those with ischaemic ulcers was also assessed using the Chi-squared and Fischer exact tests (software used was GB STAT v6.5). The odds ratio was also determined with confidence intervals calculated at 95% level of certainty. Also, levels of significance of causes of death between DFU patients and control patients and between neuropathic and ischaemic ulcer patients on both death certification and postmortem results were calculated and expressed as *P* values in Tables 2, 3, and 4.

## 3. Results

We identified 268 patients who no longer attended the diabetic foot clinic between April 1989 and January 2000. There were 243 confirmed deaths in DFU patients who attended the clinic during the above period. The remaining 25 subjects with DFU whose deaths could not be confirmed or information on cause of death was unobtainable were excluded from the study. Overall, loss to follow-up during this period was 9.3%. The DFU group consisted of 147 (60.5%) male patients and 96 (39.5%) female patients with an age range of 30 to 95 years. Of these, 187 had ischaemic ulcers and 56 had neuropathic ulcers as defined in Materials and Methods. Death certificates confirmed that 187 DFU (77%) subjects died in hospital.

A control group of 121 deceased diabetic patients without foot ulceration was identified from the general diabetic clinic at King's College Hospital. These were patients who attended the clinic between March 1990 and March 2000. Of the 139 patients who no longer attended the diabetic clinic, 121 patients had confirmed deaths and causes of deaths were obtained as for the ulcerated group. In 12.9%, the cause of death could not be obtained. The control group comprised 54 (44.6%) male patients and 67 (55.4%) female patients with an age range of 44 to 92 years. The majority of the control patients, 103 (85%), died in hospital, which was comparable to the DFU group. Data regarding the presence of neuropathy or ischaemia was not available for the control group. The characteristics of both DFU and control group patients are summarised in Table 1. There was no significant difference apart from the proportion of male patients which was significantly higher in the DFU group compared with the controls.

In addition to death certification, 80 (33%) DFU subjects and 30 (25%) control patients had postmortem examination.

### 3.1. Subjects Lost to Follow-Up in That the Cause of Death Could Not Be Obtained

There were 25 DFU patients and 18 control patients whose cause of death could not be acquired. These patients may have moved from one region to another within the UK or possibly emigrated abroad. There are no reliable procedures for confirmation of death in this group, as deaths have not been notified to the Central Register in England and Wales. It was not possible to obtain the death certificate (or postmortem results) for these subjects as there was no trace at the Family Records Centre or the local registry office. We therefore excluded these subjects from our study.

The DFU group consisted of 12 (48%) male patients and 13 (52%) female patients. The mean age was 73.35 ± 9.32 years. There was no significant difference between patients whose cause of death was ascertained and those whose cause of death was not obtained for both age (*P* = 0.174) and proportion of males to females (*P* = 0.230).

The control group consisted of 9 (50%) male and 9 (50%) female patients. Mean age was 75.88 ± 10.33 years. There was no significant difference between patients whose cause of death was ascertained and those whose cause of death was not obtained for both age (*P* = 0.107) and proportion of males to females (*P* = 0.670).

### 3.2. Causes of Mortality in DFU Patients and Control Group on Death Certification

Overall, IHD was the major immediate cause of death (stated as cause 1a, 1b, or 1c on the death certificate) in 111 (45.7%) DFU patients (Table 2). The IHD category comprised coronary atheroma and coronary artery disease which were reported as cause of death in 89/111, myocardial infarction (MI) or coronary thrombosis and occlusion in 47/111, and cardiac arrest in 5/111 death certificates. Each one of IHD subcategories may have been reported alone or in combination with one or more of the other subcategories. The “other cardiac causes” category comprised deaths due to hypertensive heart failure, myocardial degeneration and fibrosis, and haemopericardium.

The results were similar in the control group with IHD accounting for 55 (45.5%) of all deaths. In the IHD category, coronary atheroma and coronary artery disease were stated in 45/55, MI or coronary thrombosis and occlusion in 32/55, and cardiac arrest in 1/55 on death certificates.

There was no significant difference in the other causes of death between DFU patients and controls apart from a significantly higher number of deaths in the control group from cancer (19/121) compared with the DFU group (20/243) (odds ratio 2.077, 95% confidence interval 1.062–4.060, and *P* = 0.032) (Table 2).

### 3.3. Causes of Mortality in DFU and Control Groups on Postmortem Examination

Postmortem examination results were obtained for 80 DFU patients. The results confirmed that IHD was the commonest immediate cause of death and the proportion had risen to 62.5% of all DFU deaths compared with 45.7% from death certification (Table 2). The postmortem results in 30 control group patients showed a slightly higher proportion of IHD deaths, 70.0% (21) compared to 62.5% (50) for the DFU group. This difference, however, did not reach statistical significance (*P* = 0.465) (Table 2). On postmortem examination, there was no significant difference in other causes of death including cancer between the DFU and control groups.

### 3.4. Ulcer Type and Cause of Death as Determined by Postmortem and on Death Certification

To examine the relationship between cause of death and ulcer type, patients were stratified into two categories: neuropathic and ischaemic foot ulcers. Of the 80 DFU patients who had postmortem examination, 24 were neuropathic and 56 were ischaemic DFUs. The postmortem data demonstrated that mortality from IHD was significantly higher in the neuropathic group compared to the ischaemic group (79.2% versus 55.4%, resp.) (odds ratio 3.064, 95% confidence interval 1.003–9.366, and *P* = 0.049) (Table 3). Specifically, there was a significantly increased mortality from myocardial infarction/coronary thrombosis in the neuropathic group (13/24) compared with ischaemic group (13/56) (odds ratio 3.909, 95% confidence interval 1.417–19.783, and *P* = 0.009) and also a trend to increased mortality from CAD/atherosclerosis in the neuropathic group (16/24) compared with ischaemic group (24/56) (odds ratio 2.667, 95% confidence interval 0.981–7.250, and *P* = 0.055) (Table 4). There was no other significant difference in the cause of death between patients with neuropathic and ischaemic ulcers.

Regarding patients whose death certificate was available, 56 had neuropathic and 187 had ischaemic ulcers. Death certificate data showed that mortality from myocardial infarction/coronary thrombosis was significantly increased in the neuropathic group (17/56) compared with the ischaemic group (30/187) (odds ratio 2.281, 95% confidence interval 1.434–4.551, and *P* = 0.0193) (Table 4). There was no other significant difference in the cause of death between patients with neuropathic and ischaemic ulcers.

### 3.5. Age at Death

We further determined the mean age at death in patients who had a postmortem examination. The mean age at death from IHD in the DFU group was five years lower than that in the control group (68.2 ± 8.7 years versus 73.1 ± 7.96 years, *P* = 0.015). In the neuropathic ulcer group, the mean age at death from IHD on postmortem was 67.9 ± 8.5 years compared with a mean age at death for the ischaemic ulcer patients of 68.5 ± 8.9 years (*P* = 0.407). The mean age at death for all causes combined (on death certificates and postmortem examinations) showed no significant difference between the DFU group and the control patients, 71.2 ± 11.1 years versus 72.8 ± 10.1 years (*P* = 0.091). The mean age at death from IHD (on death certificates and postmortems combined) in the DFU group was 69.5 ± 9.5 versus 72.6 ± 8.3 years for the control group (*P* = 0.051).

## 4. Discussion 

This 11-year retrospective audit directly defines the precise causes of death in DFU patients. Postmortem results were examined to avoid inaccuracies as can occur with death certification [9]. The results confirmed IHD as the major cause of death in DFU patients. In particular, death from myocardial infarction was significantly higher in the neuropathic group compared with the ischaemic group both on death certification and postmortem findings. Our results concur with other studies which showed the increased mortality risk to be ascribed to cardiovascular disease in particular IHD [10, 11]. In addition, our study established that the risk of premature mortality from IHD is greater in patients who develop neuropathic ulceration. We have earlier addressed the role of IHD in the increased mortality rate associated with DFU through the application of a proportionate model of the DFU population [7]. We used the model to show that a 25% reduction in the number of neuropathic DFU patients dying earlier than nonulcerated subjects eliminated increased mortality. The link between neuropathic foot ulceration and the excess mortality from IHD demands further clarification.

Latest evidence shows that DFU has a major independent influence on lower extremity amputation and mortality risk which is quite apart from other baseline complications [12]. Our postmortem data indicated a significantly high frequency of IHD in neuropathic ulcer patients (79.2%) compared to the ischaemic group (55.4%). This is in keeping with the study of Boyko et al. stating that, amongst patients with diabetic foot ulcers who died, 64% of these ulcers were judged to be due to neuropathy, and mortality was independent of macrovascular disease as measured by ankle-arm index [1]. More recent studies have confirmed that mortality in patients presenting with neuropathic ulcers was unexpectedly high with an average 14-year reduction in life expectancy related to neuropathy whether an ulcerated neuropathic or a Charcot foot [13].

### 4.1. Possible Reasons for Link between Neuropathy and Death 

#### 4.1.1. Peripheral Somatic Neuropathy

Large nerve fibre dysfunction related to diabetes, as measured by vibration perception threshold, is strongly linked with a high risk of foot ulceration [14]. It also predicts amputation and mortality even in young type 1 diabetes patients and is associated with increased cardiovascular risk [15]. Because of the established strong association between lower extremity neuropathy and diabetic foot lesions, death related to diabetic foot problems (including ulceration) has been used as an estimate of mortality associated with peripheral neuropathy [16]. In a 14-year observational study, the main microvascular complications of diabetes (peripheral sensory neuropathy and nephropathy) were associated with increased mortality in diabetic patients although abnormal vibration threshold was more strongly associated with increased mortality than other microvascular complications [17].

#### 4.1.2. Autonomic Neuropathy

Patients with large fibre neuropathy also have evidence of small fibre neuropathy including autonomic neuropathy, which is associated with increased mortality from cardiovascular disease, particularly sudden cardiac death [18]. Peripheral autonomic neuropathy (small fibre) is associated with the development of foot ulceration in diabetic subjects. Measures of peripheral autonomic neuropathy in terms of peripheral vascular, cardiovascular, and neurophysiological measurements are worse in neuropathic ulcer patients when compared with nonulcerated patients [19]. Diabetic patients with autonomic dysfunction affecting cardiac efferent sympathetic signals have impaired sympathetically mediated dilation of coronary resistance vessels and the severity is related to the degree of sympathetic dysfunction. Impaired dilation of these vessels can lead to myocardial ischaemia and left ventricular dysfunction, even in the absence of overt atherosclerosis [20, 21]. Silent ischaemia is significantly more common in diabetic men with autonomic neuropathy than in those without as it prevents the development of angina pain. Evidence of fresh infarction may not always be found at postmortem in sudden deaths in patients with autonomic neuropathy [22].

#### 4.1.3. Neuropathy and Calcification

Neuropathy is also closely linked to calcification of vascular smooth muscle, a process thought to be mediated by receptor activator of nuclear factor kappa B ligand (RANK-L)/osteoprotegerin signalling pathway implicated in coronary and peripheral vascular disease. Vascular calcification in diabetic neuropathy may be a significant factor in increased cardiovascular risk in neuropathic ulcerated patients independent of autonomic neuropathy and cardiac denervation [23]. Therapeutic options targeting these emerging pathways may help modulate macrovascular complications and have a beneficial effect on cardiovascular outcomes in this population of diabetes patients [24].

Neuropathy may also be a marker of associated nephropathy which is a well-established risk factor for cardiovascular death. Microalbuminuria, an independent predictor of progressive nephropathy, is associated with endothelial damage and reflects atherosclerotic disease and vascular dysfunction and has also been shown to be strongly associated with the development of diabetic foot ulcers in type 2 diabetic patients [25]. Patients with diabetic nephropathy have a high frequency of autonomic neuropathy and both factors are associated with and contribute independently to the risk of silent ischaemia [26]. Cardiac autonomic neuropathy is also an independent risk factor for cardiovascular morbidity and mortality in type 1 diabetic patients with nephropathy [27]. Moreover, survival after amputation is lower in diabetic patients with chronic kidney disease and those on dialysis and this may be related to the severity of neuropathy amongst other comorbidities in these patients [28]. Autonomic neuropathy, however, is difficult to diagnose on postmortem studies, let alone ascertain the degree of severity of autonomic dysfunction.

Some of the excess mortality has also been thought to be due to uncontrolled sepsis [12]. We have shown that foot infection with* Staphylococcus aureus*, which is a very common offender in DFUs, increases the mortality rate 2.6 times compared to those without* Staphylococcus* infection [29]. It is postulated that* Staphylococcus aureus* could increase the risk of mortality through a cytokine response, which might cause plaque rupture and subsequent death from myocardial infarction. Strong evidence exists for the importance of a vagus nerve-mediated pathway in controlling cytokine production essential for preventing pathological inflammation [30]. The activation of the efferent vagus nerve stimulates the release of acetylcholine which inhibits the release of tumor necrosis factor (TNF), high mobility group box-1 (HMGB1), and other proinflammatory cytokines from resident tissue macrophages (the cholinergic anti-inflammatory pathway) without affecting the production of anti-inflammatory cytokines. This inhibits excessive systemic inflammation and protects against endotoxaemia and ischaemia reperfusion injury [31, 32]. This process is attenuated in autonomic dysfunction resulting in decreased vagus nerve anti-inflammatory output, which might be associated with loss of tonic suppression of inflammatory processes [30]. Thus neuropathic patients may be at increased risk of more flagrant inflammation and more extensive endothelial dysfunction of their coronary arteries.

Potential confounding factors of the relationship between cause of death and ulcer type include ageing. Neuropathy is frequently associated with ageing [33]. The increased mean age of this sample increases the possibility of IHD being a late diabetic complication. On the other hand, published data show that diabetes* per se* is associated with excess mortality, even in an area with high background death rates from cardiovascular disease [34]. More recently, studies have implicated QTc prolongation in type 2 diabetes patients with foot ulcers in the excess mortality observed in these patients [35].

### 4.2. Strengths and Limitations of Study

The use of our local nonulcerated diabetic population, as opposed to the general population, as a control group minimised bias in the estimates of relative mortality rates. The classification of causes of death facilitated comprehensive comparisons of all causes of mortality between the two groups with and without foot ulcers. Notwithstanding the loss to follow-up rate of 9.3% in acquiring the cause of death in DFU patients, the data collection method allowed for representation of most of the population in the South East of England referred with foot ulceration to this centre with no specific groups excluded. It was not possible to acquire death certificates in 25 of the DFU group and 18 of the controls. However, there was no significant difference between patients whose cause of death was ascertained and those whose cause of death was not obtained in both DFU and control patients for both age and proportion of males to females. This retrospective study looks primarily at the causes of death in DFU patients. It was not feasible to look at lifestyle factors or analyse the events preceding their deaths. It was also not possible to adjust for independent risk factors for IHD in the neuropathic group, for example, lipid profile and smoking habits. Other comorbid factors such as the presence of nephropathy have not been looked at. It is accepted that the deaths took place from 1989 up to 2000. Since then, newer diabetes treatments may have improved cardiovascular outcome. However, despite modern treatments, mortality of diabetic foot patients is still high [36].

Controlling for DFU patients also poses a significant problem as the general population does not provide specific controls, which in the case of DFU patients is difficult to determine. Although the DFU group had a higher proportion of males compared with the control group, the frequency of the causes of death was similar. While death certification showed a significantly higher number of deaths in the control group from cancer compared with the DFU group, this was not confirmed on postmortem studies. Data was not available for the presence of ischaemia or neuropathy in the control group. This emphasises the importance of a prospective study to account more accurately for levels of ischaemia and neuropathy and possibly the rate of change of these baseline categorisations.

## 5. Conclusion

IHD has long been recognised as an increased risk for individuals with diabetes. Our results suggest that ulcer type influences the incidence of IHD. This study provides strong evidence to reiterate its importance as a factor, placing neuropathic diabetic foot ulcer patients at a considerable risk of premature death. We have attempted to elucidate the mechanisms implicating IHD in the excess premature mortality in neuropathic DFU patients. The survival benefits of introducing an aggressive cardiovascular risk management programme in DFU clinics have also been proven and this can direct future implementation of national programmes [37].

## Figures and Tables

**Table 1:** Characteristics of diabetic foot ulcer patients and controls.

Characteristic	DFU (*n* = 243)	Controls (*n* = 121)	*P*
Mean age in years	71.2 ± 11.1	72.8 ± 10.1	0.090
Sex			
Males	147 (60.5%)	54 (44.6%)	0.005
Females	96 (39.5%)	67 (55.4%)	
Type of diabetes			
Type 1	28 (11.5%)	12 (10 %)	0.3
Type 2	169 (69.5%)	109 (90 %)	
Unknown	46 (19%)	0	
Place of death			
Hospital	187 (77%)	103 (85%)	0.07
Home	56 (23%)	18 (14.9%)	

**Table 2:** Causes of death in diabetic foot ulcer patients and control group on death certification and postmortem examination.

Cause of death	DFU pts on DC (*n* = 243)	Control pts on DC (*n* = 121)	*P*	DFU pts on PM (*n* = 80)	Control pts on PM (*n* = 30)	*P*
Ischaemic heart disease	111 (45.7%)	55 (45.5%)	0.968	50 (62.5%)	21 (70%)	0.465
(i) CAD/atherosclerosis	89	45	0.976	47	16	0.609
(ii) MI/coronary thrombosis	47	32	0.123	27	11	0.775
(iii) Cardiac arrest	5	1	0.401	0	0	0.629
Other cardiac causes	13 (5.3%)	7 (5.8%)	0.864	7 (8.8%)	3 (10%)	0.839
Bronchopneumonia	39 (16 %)	21 (17.4%)	0.752	3 (3.8%)	4 (13.3%)	0.085
Cancer	20 (8.2%)	19 (15.7%)	0.033	2 (2.5%)	1 (3.3%)	0.812
Cerebrovascular accidents	11 (4.5%)	6 (5%)	0.854	1 (1.3%)	0	0.932
Septicaemia	10 (4.1%)	6 (5%)	0.712	0	0	0.629
Renal failure	10 (4.1%)	3 (2.5%)	0.433	0	0	0.629
Pulmonary thromboembolic disease	8 (3.3%)	1 (0.8%)	0.187	7 (7.5%)	1 (3.3%)	0.349
Gastrointestinal bleeding	5 (2.1%)	0	0.245	2 (0.8%)	0	0.671
Chronic obstructive pulmonary disease	3 (1.2%)	1 (0.83%)	0.727	1 (1.25%)	0	0.932
Ruptured aortic aneurysm	2 (0.8%)	1 (0.83%)	0.997	2 (2.5%)	0	0.671
Other causes	11 (4.4%)	1 (0.83%)	0.098	5 (6.25%)	0	0.318

DC: death certification, PM: postmortem, CAD: coronary artery disease, and MI: myocardial infarction.

**Table 3:** Association of ischaemic heart disease with ulcer type on postmortem examination.

Ulcer type	Death from IHD	Death from other causes	Total	*P*
Neuropathic	19 (79.2%)	5 (20.8%)	24	0.049
Ischaemic	31 (55.4%)	25 (44.6%)	56	

Total	50	30	80	

**Table 4:** Deaths and causes of death in neuropathic and ischaemic ulcer patients on death certification and postmortem examination.

Cause of death	Neuropathic on DC (*n* = 56)	Ischaemic on DC (*n* = 187)	*P*	Neuropathic on PM (*n* = 24)	Ischaemic on PM (*n* = 56)	*P*
Ischaemic heart disease	28 (50%)	83 (44.4%)	0.460	19 (79.2%)	31 (55.4%)	0.049
(i) CAD/atherosclerosis	23	57	0.141	16	24	0.055
(ii) MI/coronary thrombosis	17	30	0.019	13	13	0.009
(iii) Cardiac arrest	0	5	0.519	0	0	0.116
Other cardiac causes	1 (1.8%)	12 (6.4%)	0.188	0	7 (12.5%)	0.176
Bronchopneumonia	7 (12.5%)	32 (17.1%)	0.411	1 (4.2%)	2 (3.6%)	0.082
Cancer	8 (14.3%)	12 (6.4%)	0.067	0	2 (3.6%)	0.606
Pulmonary thromboembolic disease	1 (1.8%)	7 (3.7%)	0.482	1 (4.2%)	5 (8.9%)	0.469
Cerebrovascular accident	1 (1.8%)	10 (5.4%)	0.285	0	1 (1.8%)	0.865
Septicaemia	2 (3.6%)	8 (4.3%)	0.816	0	0	0.678
Chronic obstructive pulmonary disease	0	3 (1.6%)	0.616	0	2 (3.6%)	0.606
Renal failure	4 (7.1%)	6 (3.2%)	0.205	0	0	0.678
Gastrointestinal bleeding	1 (1.8%)	4 (2.1%)	0.870	1 (4.2%)	0	0.232
Ruptured abdominal aortic aneurysm	1 (1.8%)	1 (0.5%)	0.392	1 (4.2%)	1 (1.8%)	0.544
Other	2 (3.6%)	9 (4.8%)	0.701	1 (4.2%)	4 (7.1%)	0.619

DC: death certification, PM: postmortem, CAD: coronary artery disease, and MI: myocardial infarction.

## Abbreviations

DFU: Diabetic foot ulcer
IHD: Ischaemic heart disease.

## References

[1] Boyko E. J., Ahroni J. H., Smith D. G., Davignon D. (1996). Increased mortality associated with diabetic foot ulcer. *Diabetic Medicine*.

[2] Nelzen O., Bergqvist D., Lindhagen A. (1997). Long-term prognosis for patients with chronic leg ulcers: a prospective cohort study. *European Journal of Vascular and Endovascular Surgery*.

[3] Challeton J. P., Letanoux M., Melki J. P., Mourad J. J., Priollet P. (1993). Le pied diabétique: pronostic dans une série de 75 patients. *La Revue de Médecine Interne*.

[4] Klamer T. W., Towne J. B., Bandyk D. F., Bonner M. J. (1987). The influence of sepsis and ischaemia on the natural history of the diabetic foot. *The American Surgeon*.

[5] Moulik P. K., Mtonga R., Gill G. V. (2003). Amputation and mortality in new-onset diabetic foot ulcers stratified by etiology. *Diabetes Care*.

[6] Iversen M. M., Tell G. S., Riise T. (2009). History of foot ulcer increases mortality among individuals with diabetes: ten-year follow-up of the Nord-Trøndelag health study, Norway. *Diabetes Care*.

[7] Chammas N. K., Hill R. L. R., Foster A. V. M., Edmonds M. E. (2002). Is neuropathic ulceration the key to understanding increased mortality due to ischaemic heart disease in diabetic foot ulcer patients? A population approach using a proportionate model. *Journal of International Medical Research*.

[8] Edmonds M. E., Blundell M. P., Morris M. E., Thomas E. M., Cotton L. T., Watkins P. J. (1986). Improved survival of the diabetic foot: the role of a specialised foot clinic. *Quarterly Journal of Medicine*.

[9] Mühlhauser I., Sawicki P., Blank M., Overmann H., Richter B., Berger M. (2002). Reliability of causes of death in persons with type I diabetes. *Diabetologia*.

[10] Hansson C., Andersson E., Swanbeck G. (1987). A follow-up study of leg and foot ulcer patients. *Acta Dermato-Venereologica*.

[11] Brownrigg J. R. W., Davey J., Holt P. J. (2012). The association of ulceration of the foot with cardiovascular and all-cause mortality in patients with diabetes: a meta-analysis. *Diabetologia*.

[12] Martins-Mendes D., Monteiro-Soares M., Boyko E. J. (2014). The independent contribution of diabetic foot ulcer on lower extremity amputation and mortality risk. *Journal of Diabetes and its Complications*.

[13] Van Baal J., Hubbard R., Game F., Jeffcoate W. (2010). Mortality associated with acute charcot foot and neuropathic foot ulceration. *Diabetes Care*.

[14] Young M. J., Breddy J. L., Veves A., Boulton A. J. M. (1994). The prediction of diabetic neuropathic foot ulceration using vibration perception thresholds: a prospective study. *Diabetes Care*.

[15] Elliott J., Tesfaye S., Chaturvedi N. (2009). EURODIAB prospective complications study group. *Diabetes Care*.

[16] Weng C., Coppini D. V., Mozzakka N., Sönkson P. H. (1996). Deaths related to diabetic foot problems as an estimate of mortality associated with peripheral neuropathy. *Diabetic Medicine*.

[17] Coppini D. V., Bowtell P. A., Weng C., Young P. J., Sönksen P. H. (2000). Showing neuropathy is related to increased mortality in diabetic patients—a survival analysis using an accelerated failure time model. *Journal of Clinical Epidemiology*.

[18] Ewing D.-J., Campbell I.-W., Clarke B. F. (1976). Mortality in diabetic autonomic neuropathy. *The Lancet*.

[19] Gilmore J. E., Allen J. A., Hayes J. R. (1993). Autonomic function in neuropathic diabetic patients with foot ulceration. *Diabetes Care*.

[20] Di Carli M. F., Bianco-Batlles D., Landa M. E. (1999). Effects of autonomic neuropathy on coronary blood flow in patients with diabetes mellitus. *Circulation*.

[21] Scognamiglio R., Avogaro A., Casara D. (1998). Myocardial dysfunction and adrenergic cardiac innervation in patients with insulin-dependent diabetes mellitus. *Journal of the American College of Cardiology*.

[22] O'Sullivan J. J., Conroy R. M., MacDonald K., McKenna T. J., Maurer B. J. (1991). Silent ischaemia in diabetic men with autonomic neuropathy. *British Heart Journal*.

[23] Jeffcoate W. (2004). Vascular calcification and osteolysis in diabetic neuropathy—is RANK-L the missing link?. *Diabetologia*.

[24] Ndip A., Wilkinson F. L., Jude E. B., Boulton A. J. M., Alexander M. Y. (2014). RANKL–OPG and RAGE modulation in vascular calcification and diabetes: novel targets for therapy. *Diabetologia*.

[25] Guerrero-Romero F., Rodríguez-Morán M. (1998). Relationship of microalbuminuria with the diabetic foot ulcers in type II diabetes. *Journal of Diabetes and its Complications*.

[26] Beck M. O., Silveiro S. P., Friedman R., Clausell N., Gross J. L. (1999). Asymptomatic coronary artery disease is associated with cardiac autonomic neuropathy and diabetic nephropathy in type 2 diabetic patients. *Diabetes Care*.

[27] Astrup A. S., Tarnow L., Rossing P., Hansen B. V., Hilsted J., Parving H.-H. (2006). Cardiac autonomic neuropathy predicts cardiovascular morbidity and mortality in type 1 diabetic patients with diabetic nephropathy. *Diabetes Care*.

[28] Lavery L. A., Hunt N. A., Ndip A., Lavery D. C., Van Houtum W., Boulton A. J. M. (2010). Impact of chronic kidney disease on survival after amputation in individuals with diabetes. *Diabetes Care*.

[29] Mantey I., Hill R. L., Foster A. V., Wilson S., Wade J. J., Edmonds M. E. (2000). Infection of foot ulcers with *Staphylococcus aureus* associated with increased mortality in diabetic patients. *Communicable Disease and Public Health*.

[30] Pavlov V. A., Tracey K. J. (2006). Controlling inflammation: the cholinergic anti-inflammatory pathway. *Biochemical Society Transactions*.

[31] Bernik T. R., Friedman S. G., Ochani M. (2002). Cholinergic anti-inflammatory pathway inhibition of tumor necrosis factor during ischemia reperfusion. *Journal of Vascular Surgery*.

[32] Czura C. J., Friedman S. G., Tracey K. J. (2003). Neural inhibition of inflammation: the cholinergic anti-inflammatory pathway. *Journal of Endotoxin Research*.

[33] Adler A. I., Boyko E. J., Ahroni J. H., Stensel V., Forsberg R. C., Smith D. G. (1997). Risk factors for diabetic peripheral sensory neuropathy. Results of the Seattle Prospective Diabetic Foot Study. *Diabetes Care*.

[34] Roper N. A., Bilous R. W., Kelly W. F., Unwin N. C., Connolly V. M. (2001). Excess mortality in a population with diabetes and the impact of material deprivation: longitudinal, population based study. *British Medical Journal*.

[35] Fagher K., Löndahl M. (2013). The impact of metabolic control and QTc prolongation on all-cause mortality in patients with type 2 diabetes and foot ulcers. *Diabetologia*.

[36] Morbach S., Furchert H., Gröblinghoff U. (2012). Long-term prognosis of diabetic foot patients and their limbs: amputation and death over the course of a decade. *Diabetes Care*.

[37] Young M. J., McCardle J. E., Randall L. E., Barclay J. I. (2008). Improved survival of diabetic foot ulcer patients 1995–2008. *Diabetes Care*.

